# A Rare Complication of Congenital Afibrinogenemia: Bone Cysts

**DOI:** 10.4274/tjh.2015.0382

**Published:** 2017-06-01

**Authors:** Ali Fettah, Dilek Gürlek Gökçebay, Vildan Çulha, Neşe Yaralı, Bahattin Tunç, Namık Özbek

**Affiliations:** 1 Ankara Children’s Hematology and Oncology Research Hospital, Clinic of Pediatric Hematology, Ankara, Turkey

**Keywords:** Afibrinogenemia, Bone cysts, child, Rare

A 12-year-old male patient diagnosed with congenital afibrinogenemia presented to our center with pain, swelling, and ecchymosis in his leg after trauma. His past medical history revealed that he had been diagnosed with afibrinogenemia shortly after birth because of umbilical bleeding. Laboratory tests at admission revealed prolonged prothrombin time and activated partial thromboplastin time, and almost undetectable fibrinogen levels. A bone scan and radiograms of both legs showed multiple cystic lesions in the tibiae (Figure 1). Magnetic resonance imaging (MRI) of the legs also showed multicystic lesions with septae formation involving metaphyseal-diaphyseal junctions (Figure 2).

Bone cysts, one of the rare complications of afibrinogenemia, frequently appear in the contiguity of the cortex or trabeculae in the diaphysis of long bones, particularly the femora, tibiae, and humeri, and should be considered in patients who suffer rheumatic pains of the extremities [1,2]. Intraosseous hemorrhage, usually at the entrance of the nutrient artery, causes intraosseous cysts. Large cysts, especially in weight-bearing bones, may cause pathological fractures [2,3]. Whole-body MRI might be useful to evaluate the lesions. We want to emphasize the importance of on-demand therapy and MRI in determining bone cysts. However, the role of secondary prophylaxis needs to be evaluated.

## Figures and Tables

**Figure 1:**
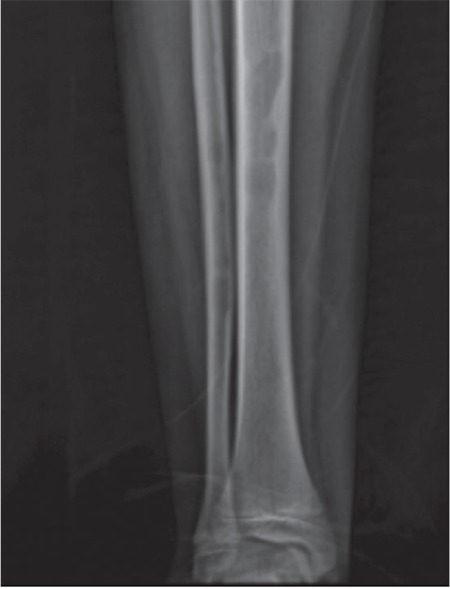
Direct radiography of the tibia.

**Figure 2:**
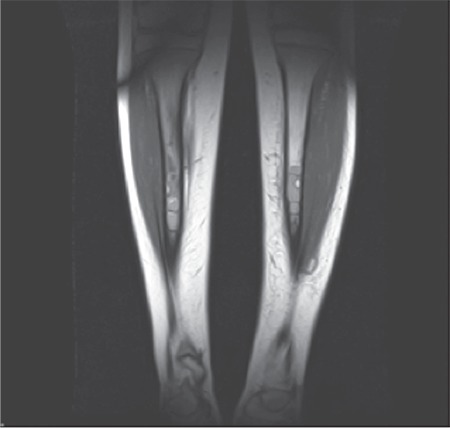
Magnetic resonance imaging of the lower extremities.
